# Clinical perspectives on sampling and processing approaches for the management of infection in diabetic foot ulceration: A qualitative study

**DOI:** 10.1111/iwj.14912

**Published:** 2024-06-10

**Authors:** Erica di Martino, Elizabeth Andrea Nelson, Jane Elizabeth Nixon, David Alexander Russell, Anna Louise Goodman, Sanjeev Rasiklal Mehta, Frances Game

**Affiliations:** ^1^ Faculty of Medicine and Health University of Leeds Leeds UK; ^2^ School of Health and Life Sciences Glasgow Caledonian University Glasgow UK; ^3^ Centre for Clinical Infection and Diagnostics Research (CIDR), Guy's and St Thomas NHS Trust and King's College London MRC Clinical Trials Unit at UCL London UK; ^4^ Ealing Hospital London North West University Healthcare NHS Trust London UK; ^5^ Research and Development Department University Hospitals of Derby and Burton NHS Foundation Trust Derby UK

**Keywords:** diabetic foot, expert testimony, polymerase chain reaction, qualitative research

## Abstract

Diabetic foot ulcers (DFUs) often become infected and are treated with antimicrobials, with samples collected to inform care. Swab samples are easier than tissue sampling but report fewer organisms. Compared with culture and sensitivity (C&S) methods, molecular microbiology identifies more organisms. Clinician perspectives on sampling and processing are unknown. We explored clinician perspectives on DFU sampling—tissue samples/wound swabs—and on processing techniques, culture and sensitivity or molecular techniques. The latter provides information on organisms which have not survived transport to the laboratory for culture. We solicited feedback on molecular microbiology reports. Qualitative study using semi‐structured interview, with analysis using a Framework approach. CODIFI2 clinicians from UK DFU clinics. Seven consultants agreed to take part. They reported, overall, a preference for tissue samples over swabbing. Clinicians were not confident replacing C&S with molecular microbiology as the approach to reporting was unfamiliar. The study was small and did not recruit any podiatrists or nurses, who may have discipline‐specific attitudes or perspectives on DFU care. Both sampling approaches appear to be used by clinicians. Molecular microbiology reports would not be, at present, suitable for replacement of traditional culture and sensitivity.

## 1 INTRODUCTION

Infection of diabetic foot ulcers (DFU) is a significant cause of morbidity and mortality in people with diabetes.^1^ DFUs may become clinically infected, and these diabetes‐related foot infections (DFIs) are associated with increasing morbidity, more frequent healthcare provider visits, more complex and frequent wound care, antimicrobial therapy, surgical procedures and high healthcare costs.^2^ Of particular importance, DFIs remain the most frequent diabetes‐related complications requiring hospitalisation and the most common precipitating events leading to lower extremity amputation. Prompt treatment with an empiric choice of antimicrobial therapy is crucial to avoid complications, and this is later tailored, upon receipt of clinical microbiology results, to the specific microorganisms causing the infection and their antibiotic resistance profile.

The gold‐standard test to guide treatment involves taking a tissue or swab sample from the infected ulcer and then using standard microbiology techniques to grow the microorganisms and test their susceptibility to a range of antibiotics.^3^ This methodology is time consuming and requires 2–3 days, sometimes more, before results are available. In addition, as it involves growing the bacteria, it can only identify live microorganisms which survive collection and transport and are in a biological state to grow in standard culture conditions. Recently, molecular techniques have become available, which identify the presence of bacteria through the detection of their genetic fingerprint by polymerase chain reaction (PCR) and can potentially provide results in <1 h^4^, ^5^ from receipt of the sample in the laboratory. A faster result might allow clinicians to prescribe tailored antibiotic therapy at the initial patient visit or the next day, with a likely potential improvement in the precision of antimicrobial prescribing and hence in clinical outcome. A second advantage of molecular techniques is that they do not rely on the presence of viable bacteria, therefore reducing the likelihood of false‐negative results in patients who have already started a course of antibiotics. Finally, it is hypothesised that the use of rapid and precise diagnostic testing may reduce the use of broad‐spectrum antibiotics, which contribute to the development of antimicrobial resistance, an emerging threat to global health. Potential disadvantages are that reports of dead organisms may lead to overtreatment of organisms no longer relevant and the antibiotic use may become broader to include coverage for non‐pathogenic, colonising and/or environmental organisms.

Molecular techniques are becoming more widely available, especially as the number of microorganisms that they can detect increases. However, most clinicians are not yet familiar with the reports produced from these diagnostic techniques, which may identify many more species than the standard microbiology methods, and provide the results in a different format from the semi‐quantitative approaches used to date, for example, describing prevalence as +, ++ or +++, rather than as a proportion of organisms, as is the case with PCR molecular microbiology reports. Hence the utility of the report depends upon not only the detection of the organisms, but also the application of specialist knowledge by the microbiologist to aid interpretation, for example, by noting thresholds for potential relevance. Therefore, it is uncertain how clinicians will interpret these new reports and how they might use them to inform prescribing practice.

As part of the COncordance in DIabetic Foot Infection 2 (CODIFI2) clinical trial (https://www.isrctn.com/ISRCTN74929588), we undertook a qualitative sub‐study to evaluate the perspectives of healthcare professionals (HPs) on PCR microbiology techniques, with focus on their utility and ease of interpretation, whether they perceive them as replacements for or complementary to the traditional microbiology techniques of culture and sensitivity testing, and what type of support clinicians would need to become confident users of PCR microbiology reports. Ethical approval was obtained from the School of Healthcare, University of Leeds, Ethics Committee.

## 2 METHODS

### 2.1 Study design

Qualitative study using in‐depth semi‐structured interviews with health professionals (HPs) who were independent prescribers involved in the clinical management of patients with DFUs at UK NHS sites taking part in the CODIFI2 trial. This was a pre‐defined sub‐study to complement the trial of sampling of infected diabetic foot ulcers (swab vs. tissue), and comparison of the reports from microbiology using culture and sensitivity versus genetic approaches (PCR).

### 2.2 Sample identification and recruitment

Principal investigators of the participating CODIFI2 trial sites were asked to circulate the details of the study to HPs within their teams. Staff members from the CODIFI2 study sites, whose contact details were already held for the purposes of trial communication, were also sent information directly. Sites which successfully recruited at least one participant to CODIFI2 were approached first, followed by all other sites which opened to recruitment. One single reminder was sent 2 weeks after the initial approach. Snowball sampling was also used to identify further potential participants. We estimated a sample size of up to 12 participants to achieve spread (range of sites and health professional roles) and depth (understanding of the views of different individuals).^6^

### 2.3 Data collection

Following the provision of study information and obtaining informed consent, interviews were conducted, recorded and transcribed verbatim using Microsoft Teams during June 2023. Interviews used a flexible topic guide developed based on our previous research and initial conversations with members of the trial team (Table 1). Participants were asked about current practice in sampling, prescribing and following up with patients with infected DFUs, their opinions on two example PCR reports (Tables 2 and 3) and on the possible future role of molecular PCR tests in clinical practice. Each healthcare professional was interviewed once, for 20–35 min.

**TABLE 1:** Interview schedule.

	Interviewer states/asks	Prompt, if needed…
Question 1.	We are interested in understanding how to improve the assessment and management of clinically infected diabetic foot ulcers.	Do you take a sample and initiate empiric antibiotics on the same day, for example? Or do you initiate antibiotics empirically, without sampling?
	Can you briefly talk me through your approach with respect to three aspects: Microbiological sampling Prescribing antimicrobials, and Clinical follow‐up?	And what about follow‐up? Do your review at the next appointment or add an additional review appointment in a few days to assess the clinical response in conjunction with the microbiology results?
Question 2	We are currently looking at the potential role of polymerase chain reaction (PCR) microbiology to support the management of infected diabetic foot ulcers. PCR techniques may provide more rapid results, as well as detecting species that do not get picked up on conventional culture and sensitivity. In the future, we think it may be possible for clinics to get rapid PCR microbiology results for infected diabetic foot ulcer samples, which might complement or replace the current MC&S reports. Within the CODIFI2 study, we have some PCR results from infected diabetic foot ulcers. Here is a sample report for you to see the type of detail provided. We did like to get your clinical expert opinion as to the interpretation and usefulness of this type of information. Can you tell us what your impressions are of the PCR microbiology report?	What do you think about the length of the report, the number of species detailed, and the information provided about relative bacterial load (number of bacteria)? What are your impressions regarding the ease of interpretation of the report? Is there anything missing that you would like to see?
Question 3	How might we improve the reporting of our PCR reports in conjunction with microbiologists and clinicians to ensure that when they arrive at the clinical site that they would be maximally useful to you?	
Question 4.	We did like to understand how useful you would find it to have a quicker report than we can get with the current ‘culture and sensitivity’ report from your microbiology laboratory. If a PCR report was available in 12–24 h, would that make any difference to your initial management and sampling? Can you tell me why?	Would you still be minded to prescribe empirical antimicrobials initially if a PCR report was going to be available on the next day, say?
Question 5	So, if we have improved the PCR report so that it is accessible and available, what role would you see for the PCR reports in your management and assessment of infected diabetic foot ulcers? Would you see it as potentially replacing the current microbiological culture and sensitivity reports that may take three to 5 days to come back to the clinic or would you see them as complementary to their culture and sensitivity reports? In what circumstances do you feel it would be appropriate to replace culture and sensitivity with rapid, accurate PCR microbiology reporting so that we are not duplicating tests? What are the barriers to our being able to replace the slower MC&S reports with rapid PCR testing?	

**TABLE 2:** Example 1 of a molecular microbiology report.

Genus	Abundance (%)	Anaerobe	Gram
Corynebacterium	24	No	Positive
Staphylococcus	72	No	Positive
Other	4		

**TABLE 3:** Example 2 of a molecular microbiology report.

Genus	Abundance (%)	Anaerobe	Gram
Alcaligenes	21	No	Negative
Providencia	7	No	Negative
Enterobacteriaceae. unclassified	15	No	Negative
Helcococcus	8	Yes	Positive
Other	4		
Escherichia Shigella	5	No	Negative
Pseudomonas	40	No	Negative

### 2.4 Analysis

Anonymised transcripts were analysed using thematic analysis guided by a Framework Approach. This involved familiarisation with the transcripts, iterative development of codes, coding of the transcripts and identification of dominant themes.^7^ The coding and framework are reported in Table 4. Data were coded, organised and summarised into framework matrices using NVivo 1.6.1 software. Final interpretation of the data involved studying the themes, the links between the themes and relating these findings back to the research questions and relevant literature.

**TABLE 4:** Analysis coding framework.

Theme	Sub‐theme	Explanation and examples
Current practice in infected DFUs This theme includes description of current practices in the clinical management of infected DFUs, including their sampling, prescribing of antimicrobials and reviewing prescription based on microbiology results.	General practices	General statements regarding current practices in clinical management of infected DFUs, for example Ulcers are graded as mild, moderate and severe
	Specific practices regarding sampling	Specific information regarding sampling of an infected DFUs, for example How the ulcer is prepared and sampled; Preference for swab or tissue sample
	Specific practices regarding prescribing	Specific information regarding prescribing antibiotics for infected DFUs, for example Antibiotics are/are not started empirically whilst waiting for swab results Type of antibiotics prescribed
	Specific practices regarding reviewing treatment based on microbiology reports	Specific information regarding reviewing treatment based on microbiology reports, for example: Antibiotic choice is/is not reviewed based on results of swab If and when antibiotic choice is changed based on microbiology report
	Limitations with current practice	Specific examples of limitations in current practices regarding clinical management of infected DFUs, for example Turnaround time for culture and sensitivity results Uninformative culture and sensitivity results Difficulties in sampling DFUs
B Healthcare professionals' (HPs) perspectives on implementation of polymerase chain reaction (PCR) microbiology tests This theme includes HPs' opinion on the utility of PCR microbiology tests, along with their potential impact on antibiotic prescribing and role in the diagnostic pathway and any barriers to their implementation	Potential clinical benefits of PCR microbiology tests	HPs' opinion on potential benefits of more rapid microbiology results, for example More tailored antibiotic treatment Improvement in patient outcomes Reduction of risks of antibiotic resistance
	Potential impact of PCR microbiology tests in antibiotic prescribing	HPs' opinion on potential impact of more rapid microbiology results on antibiotic prescribing, for example Clinician would/would not hold off prescribing until results are available Clinician would/would not change the antibiotics prescribed based on results
	Potential role of PCR microbiology tests as adjunct or replacement of current test	HPs' opinion on whether PCR microbiology should be run in parallel or could potentially replace current culture and sensitivity tests
	Barriers to implementation	HPs perspective of barriers in implementation of PCR microbiology, for example: Costs System resistance to change Increased workload
C HPs' perspectives on PCR microbiology reports This theme includes HPs' perspective on PCR microbiology reports, encompassing strengths and weakness, suggestions for improvement and support needed for their effective use.	Strengths of the reports	Comments regarding strength of the exemplar PCR microbiology reports, for example Reporting percentage of microorganism is preferred to standard abundance information
	Limitations of the reports	Comments regarding limitations of exemplar PCR microbiology reports, for example Report format is confusing Report is too detailed Report misses important information
	General comments about the reports	General observations about the exemplar PCR microbiology reports, for example: Differences with current report highlighted but without a positive or negative connotation
	Additional information and support required to increase utility of PCR microbiology reports	Suggestions on how PCR microbiology reports could be improved, and the type of support needed for their implementation, for example: Inclusion of antibiotic sensitivities Inclusion of information on clinical relevance Guidance regarding recommended antibiotics

To ensure the credibility of the analysis, the coding and thematic framework were developed by an experienced qualitative researcher in close supervision by the wider research team. A sub‐sample of transcripts was independently reviewed by one of the trial researchers to ensure consistency and reproducibility in the interpretation and application of the coding framework.

## 3 RESULTS

### 3.1 Sample characteristics

Overall, seven qualified NHS health professionals (HPs: indicated as HP1 to 7) were interviewed. The sample included comprised medical consultants in diabetes and endocrinology, surgery, infectious diseases and medical microbiology.

### 3.2 Thematic analysis

Analysis identified three main themes: current clinical practice for infected DFUs, HPs' perspectives on PCR microbiology report, and HPs' perspectives on the implementation of PCR microbiology tests. Exemplar quotes are reported in the text, with additional supporting quotes for each theme collated in Tables 5, 6, 7.

**TABLE 5:** Further example of Healthcare professionals' (HPs) quotes relating to current clinical practice in sampling of infected Diabetic foot ulcers (DFUs), prescribing of antimicrobials and reviewing prescribed therapy based on microbiology results.

THEME: Sampling of the infected DFUs	
HP2	‘*In the clinic setting, because of course as a surgeon I might do something slightly different in theatre, but in the clinic setting I will swab it and send that for culture and sensitivities. I never biopsy an ulcer in clinic*’.
HP4	‘*What you really want is a clean sample, so that might be a tissue sample rather than a swab. It depends on the exact format of the ulcer, because you don't want to make something worse by biopsying it if it's hard to get a sample*’.
HP7	‘*Our traditional approach to that in terms of sampling would be to take a deep tissue sample rather than a wound swab if we can, so post debridement of the wounds, clean, deep tissue sample to go for microbiology. If we can't take a deep tissue sample for whatever reason, sometimes it's too painful for patients to have sampling, then we would do a swab in that instance*’.
THEME: Prescribing of antimicrobials for infected DFUs	
HP2	*‘If I thought they should start on an antibiotic, I start on our hospital protocol. [….] It depends on the severity of the infection, but the underlying philosophy is I'd give a broad spectrum and then try and narrow it down once we get results after the swabs*’
HP6	*‘All depends on the type of the diabetic foot infection and the severity of the infection. So we will give antibiotics based on our local policy empirically, whilst waiting for the culture results*’
THEME: Clinical review based on result of microbiology test	
HP5	‘*So start broad and then narrow things down once you've got the results of the culture and sensitivity*’.
HP7	‘*We don't tend to narrow down antibiotics based on microbiology for soft tissue infection because by the time we've got the susceptibilities back, most people are a significant way through their course and therefore there isn't much benefit to changing people for a few days’*
THEME: Limitations of current approach	
HP4	‘*If they're already on antibiotics, taking a swab may not be helpful because you are just selecting what's left […] if you take a swab from anywhere below the waist, you'll find colonizers [….] related to faecal contamination, which will happen to anyone [….] when tissue is dead, you get you grow gram negative organisms from it but that doesn't mean it's infected*’
HP5	‘*At the moment to get the full cultures and sensitivities may take between three and five day […] so I would have to give the patient at least five days of empirical antibiotics which may not be covering the wound*’
HP6	‘*Most of the times I have to say I don't get any samples or even if I do get samples, they're not of the best quality so when we culture it, we see a constellation of colonizing organisms. The plates will be littered with organisms*’
HP7	‘*We would ideally then bring patients back to have a look and see how they're responding to that, but they're just because of the challenges of clinic, we don't really have capacity to do that*’

**TABLE 6:** Further examples of Healthcare professionals' (HPs) quotes relating to their perspectives on potential benefits of polymerase chain reaction (PCR) microbiology tests, impact on antibiotics prescribing, role as either an adjunct or a replacement of current culture and sensitivity tests and potential barriers to implementation.

THEME: Potential clinical benefits of PCR microbiology reports	
HP1	‘*We could change the antibiotics to one that's less broad spectrum and that's important, or reduce the number of antibiotics that we're using, because that's important for antibiotic resistance*’.
HP3	‘*I think diabetic foot infection are one of the most difficult thing to treat and whatever we try, it tends to fail for a lot of patients and it's multifactorial, and the organisms that we get in the swab and the antibiotic we choose, it can often be wrong. So any development in this field and in the rapidness rapidity of the results to me will make a difference*’.
HP5	‘*It would mean that that there's less likely that you would give someone an empirical antibiotic for about 5 days before you get the culture back, there's less chance of the wound deteriorating and actually if you get the sample back the same day and particularly for inpatients, they can be started on the correct antibiotic the same day. [….] So certainly it would be useful, I think definitely for inpatients, but also for other patients*’.
THEME: Potential impact of PCR microbiology reports in antibiotics prescribing	
HP2	‘*That goes back to how severe the infection is. If it's a severe infection, no. If we were confident that we'd get an accurate indication of what to give, then yeah, I think it would be entirely appropriate to NOT give antibiotics for 24 hours while we're waiting to find out which antibiotic to give specifically*’.
HP5	‘*So in some situations, if it was a mild or moderate infection, I would hold off antibiotics until the report is back. I mean it have to do it on a case‐by‐case situation. If the patient had clear signs of systemic sepsis, so severe infection, they were hypotensive, they had rigors, of course I wouldn't withhold antibiotics, I would start them straight away, but if it's a mild or moderate infection or I feel that the patient's able hemodynamically and they're not febrile, they're not rigors, I would be happy to hold off the 24 hours until the molecular report is available*’.
HP7	‘*For most people it wouldn't stop us starting antibiotics because for soft tissue infection, you've got clinical infection and you want therefore treat that, you probably wouldn't want to hold off for 24 hours*’
THEME: Potential role of PCR microbiology tests as adjunct or replacement of current test	
HP2	‘*I think it's one of those things that you'd have to run in parallel for a while until departments became comfortable with the results. […] And once you've had a chance to audit a few times and get comfortable with it, then you could phase one or the other out*’.
HP7	‘*I suspect probably initially complementary so that people can start to get a better understanding of the molecular microbiology and compare that to the standard microbiology but, you know, ultimately, if you're gonna get a result back in 12–24 hours, then you can see that replacing standard microbiology over time as people become more comfortable with the interpretation*’.
THEME: Potential barriers to implementation of PRC microbiology tests	
HP1	‘*I can't think of any barriers on, unless you have local resistance to it, as in people would still want to continue using local tissue samples*’.
HP3	*‘The swab will continue for other parts of the body anyway, so it will be an additional job for the microbiologist to have this one*’.
HP6	‘*As you know, every trust is under sort of financial strain so it has to be sort of cost effective when you put a business case in order to introduce this test*’

**TABLE 7:** Further examples of Healthcare professionals' (HPs) quotes relating to their perspectives on the strengths and limitations of polymerase chain reaction (PCR) microbiology reports, including additional information and support required to increase their utility.

THEME: Strengths of PCR microbiology reports	
HP3	*‘Percentage is much better for me to understand’*.
HP5	*‘I think quantifying it as a percentage is good because it gives you a better idea perhaps than heavy, moderate or scanty growth’*.
THEME: Limitations of PCR microbiology reports	
HP2	‘*I think that the example 2 is too detailed and I think if I was busy, I wouldn't stop to really look at it carefully*. […] *You could almost say that both of them are too complicated, because in a sense almost don't need to know the name. If it said there's a predominance of anaerobic bacteria that are gram positive, that's all we need to know, because then we'd choose an antibiotic that is suitable for aerobic bacteria that are gram positive*’.
HP3	‘*It can be confusing for some to see whether we treat for one which is relatively small [….] around 7 or 8% and it'll be confusing for someone whether to treat it or not for that organism, while the other one takes precedence. So it may come handy after we treat the infection if the wound does not heal, then it may be worth considering treating for that too’*
HP6	‘*The caveat to that testing from our experience is it will only pick up organisms as part of their molecular targets. So you know if it has got, I don't know, 20 or 30 targets, it'll only pick up whatever targets it has. Two: you're not really going to get sensitivities like, you know, culture based methods. [….] I think one of the things that I worry about sort of diabetic foot infections is 1. difficulty in sampling, 2. Even if we get the sample, very likely we're not really going to get mono microbial organisms, it's going to be polymicrobial, so we're gonna pick up lots. [….] So I think this is useful, but I think there are limitations and largely the limitations are 1 the quality of the sampling, and 2, the sensitivities*’.
Additional information and support required to increase utility of PCR microbiology reports	
HP1	‘*What would be useful for the clinician is some guidance as to how pathogenic these organisms are. Because all you are finding is […] that it's present in the tissue. The question still is left to the clinician as to whether it's a significant pathogen or not. But that's the same with microbiological specimens that we have already as to, and this is where the microbiologist will guide you. So, what would be good in this particular instance is when the microbiology report comes up based on the PCR is that the microbiologist gives some sort of direction*’.
HP2	‘*If it came out from our own microbiology department, recommended antibiotics would be really useful. [….] So I think if you if you want to improve what you showed me so far, it should be some feedback for us from the microbiologist: what antibiotic and maybe how long we give it for etcetera, etcetera*’.
HP5	‘*I'd obviously want to know about the sensitivities to different antibiotics*’
HP7	‘*I think almost certainly these would need to come with microbiology support to start with and it may be that you know higher level of microbiology support is required ongoing, particularly if you don't get the antibiotic sensitivities, but it, as I say, it's those bugs that we don't see very often and understanding the significance of those which you know I'm presuming will improve with time, but certainly in the short to medium term I think they'd need to be more microbiology support available if this was implemented’*.

### 3.3 Current clinical practice in infected DFUs

HPs reported some variability in terms of sampling of infected DFUs. Whilst for some clinicians a swab was the routine choice, the majority of HPs preferred a deep tissue sample, when possible, but considered a swab as an acceptable option in certain circumstances, for example, patient pain, unavailability of staff with required training for tissue sampling, or the position of the ulcer. Deep tissue swabs were, overall, preferred to superficial swabs, by this group of health professionals.

What we recommend is a debridement depending upon the position of the ulcer and the foot infection. If you can debride tissue, that is probably the best. If you can't debride the tissue, and if the ulcer is over a portion of the foot whereby you can take a swab, we would recommend […] deep swabbing. (HP6)

Clinicians said that they would usually start systemic antibiotic treatment immediately, without waiting for the result of the microbiological tests. The choice of treatment would be based on the overall clinical picture including the grade (severity) of the infection, standard hospital guidelines informed by the prevalence and resistance profile of common pathogens locally, and information relating to any history of previous infections and treatment for a specific patient. Both broad and narrower spectrum antibiotics were used, depending on the specific circumstances.

When we find a patient with suspected wound infection, we do not wait for the sample to come back, we start them on treatment antibiotic. The antibiotic is based on the hospital guidelines based on the microbes locally. (HP3)

We would often treat empirically to cover common pathogens, especially Staphylococcus or Streptococcus, but if it's a recurrent ulcer we may use previous microbiological results. (HP1)

Clinicians reported that the empirically prescribed antibiotics were sometimes, but not always, changed based on the result of the microbiological sampling. The decision to change antibiotic therapy depended on clinical appraisal of patient response, as well as the type of microorganisms identified and the turnaround time of the result. Several HPs mentioned difficulty in obtaining good quality samples from infected ulcers, contamination of the sample with non‐pathogenic bacteria, and the long turnaround for current culture and sensitivity tests as major obstacles to a more tailored antibiotic treatment.

If the swab turns out to be something different to what we had given, we tend to […] change the antibiotic either face to face or send a prescription through to the patient to collect from local pharmacy or through their GP. (HP3)

We will look at the results and check that the antibiotics we chose were appropriate. Just because the bug is not covered by the antibiotic doesn't mean the antibiotic wasn't appropriate. So that has to be combined with the clinical review […] If the antibiotic worked, it's worked, the result becomes less relevant. But there are circumstances like, for example, MRSA would be a circumstance where the resistance might be highly important and might change our management. (HP4)

It can take five days for us to get our standard microbiology, so with weekends as well that could mean a week for some patients, which makes it very not worthwhile to change a 10day course if someone is responding. (HP7)

The swab results are hard hit and miss for diabetic foot disease, majority of the time it's negative or multiple organisms we don't get sensitivities. (HP3)

### 3.4 HPs' perspectives on PCR microbiology reports

When presented with two examples of PCR microbiology reports, some clinicians commented that the format requires some ‘*getting used to it*’ (HP5), with some initially finding it ‘*a bit confusing*’ (HP1) and others stating it was ‘*too detailed*’ (HP2). HPs liked the fact that the report included the prevalence of bacteria as a percentage, although some explained that it would be challenging to decide whether the less abundant microorganisms needed treatment, due to lack of information about the pathogenicity of the identified species. Most clinicians agreed that the reports could be significantly improved by including pathogenicity, species‐level information, antibiotic sensitivity information and clear microbiological guidance regarding recommended treatment. Microbiology support for the interpretation of the report and antibiotics prescribing was mentioned as a crucial element to permit the clinical application of PCR microbiology, as well as evidence of cost‐effectiveness and good test performance.

In a standard swab results, we would have the sensitive and the resistant antibiotics given by the side of the positive growth. So certainly I would prefer, any physician would prefer, the antibiotic choice next to it. (HP3)

I think the challenge would be that a number of the species that are grown aren't things that we tend to see in standard microbiology reporting, and we become more comfortable in understanding likely antibiotic susceptibilities for common bacteria that we see. […] So I think for us it would be because of lack of familiarity it'd be more difficult to know which of those might be organisms that are causing infection and which are those organisms are commensals that are present but not needing treatment and certainly in terms of antibiotic sensitivities and best antibiotics to prescribe, I would need to discuss that with a microbiologist because that's information that we just don't have enough in our heads. (HP7)

It needs a microbiologist input and proper reporting as we would for any wound culture result, you would have a microbiologist review it and write something that made sense to the clinician. The way it is written at the moment wouldn't make sense to a clinician and may cause errors. People might think something else is in there, something's in there that need to action when it doesn't. (HP4)

### 3.5 HPs' perspectives on the implementation of PCR microbiology tests

Most clinicians agreed that more rapid microbiology results, such as those potentially offered by PCR microbiology, would significantly improve patient management by allowing a more tailored antibiotic treatment regimen, thereby facilitating better patient outcomes and reducing the risk of the development of antibiotic resistance. However, one clinician disagreed on the value of more rapid and detailed microbiology reports as they might confuse the non‐microbiologist reader (HP4).

It would be beneficial in terms of reducing inappropriate use of broad‐spectrum antibiotics, and actually picking up on people who are on the wrong antibiotics. So I think, yeah, the sooner you get diabetic foot infections treated appropriately, the sooner you're likely to save them sepsis or limb loss. (HP2)

So you said that PCR is more rapid and might detect extra species. However, neither of those things are really an advantage in diabetic foot infection and we don't particularly need rapid because we're often looking at what the clinical response is. And we don't particularly want to detect extra species because it's highly likely they will be contaminants or related to ischemia. (HP4)

Even with shorter test turnaround times, however, clinicians would still prefer to prescribe antibiotics empirically whilst waiting for results, although they may hold off treatment for some patients, such as those with less severe infections.

For practical reasons for the patient, you would probably still start on them on treatment, but if you are not sure, if you think the patient can wait, it does allow you not to start on antibiotics and that is important with regard to antibiotic resistance that you're using the right antibiotic that rather than you know broad spectrum antibiotics that can cause, especially in the elderly, side effects. (HP1)

Most clinicians said that initially they would consider PCR microbiology as an additional test to be run in parallel to culture and sensitivity tests, although some thought that it could eventually replace the current test, if supported by evidence of better performance. One clinician questioned the ability to obtain samples from DFUs of sufficient quality to provide meaningful results, another pointed out that alternative better tests may be available soon. Clinicians identified system resistance to change, costs and increased workload to run an additional test as other possible barriers to implementation of PCR microbiology tests.

I think initially I see it as being complementary to it, until we all get used to it. […] if it shows that it performs better than our current microbiology reports, then certainly there there's scope for it to be used more and more in the future. (HP5)

I think it is probably going to be complementary in terms of testing. I don't think it can replace culture, but it can fit in somewhere as part of an algorithm, and I'll tell you why. Because the main thing with regards to any test is the availability of good samples. So if you can't get good samples, then whatever testing you do isn't really, you're not going to utilize the full potential of the testing. (HP6)

I think the world is moving to whole genome sequencing rather than PCR […] If you're going to change all your processes, you probably want to change to the most up to date technology, not change twice. (HP4)

## 4 DISCUSSION

This is the first study looking at health professionals' perspectives on the use of swab or tissue sampling for infected DFU, and the first to explore the utility of PCR microbiology tests as an adjunct to or replacement for the current culture and sensitivity test for the microbiological characterisation and related clinical management of infected DFUs. Most clinicians agreed that there is a clinical need to improve current diagnostic processes for infected DFUs and that a faster and more sensitive microbiology test could potentially facilitate more effective and tailored treatment. This could in turn allow better patient outcomes and contribute to antimicrobial stewardship and prevention of resistance. There was no consensus on the value of more rapid test results, however.

Our findings established that clinicians had views regards the sampling approach that related to individual patient characteristics (rather than having a blanket approach based on microbiological utility or yield). Whilst deep tissue sampling was preferred by many, swab sampling was also used.

Clinicians explained that PCR microbiology reports were only as good as the samples that were collected in the first place, and the new format of the report could be confusing to interpret. Lack of information about antibiotic sensitivity was seen as a major limitation, along with uncertainty about which of the less common species of bacteria identified by the test needed treatment. There was a general consensus between these HPs that in order to be clinically useful PCR microbiology reports will have to be accompanied by clear guidance about suggested treatment from the microbiologist. If such limitations could be overcome, most of the study clinicians agreed on the usefulness of the PCR microbiology tests as an adjunct to the current test. The clinicians' comments confirm the value placed upon expert support from microbiologists to contextualise/guide treatment, as crucial aspects of the information upon which clinical decisions on prescribing, for example, were made.

For some, PCR tests could in time replace the culture and sensitivity test, if underpinned by evidence of cost‐effectiveness and better performance, but such evidence is not yet available. Given the hypothetical clinical advantages of using molecular techniques, such as the belief that reporting more species than culture will lead to improved prescribing and better clinical outcomes, we wished to understand the clinician perspective on these novel approaches, which are becoming increasingly used, despite there being no evidence of the impact of a change in processing technique upon management in this population.^8^

Recent studies suggest that it is possible to gather resistance information for bacteria isolated from DFUs using targeted PCR techniques^9^, ^10^, ^11^ and that PCR‐based diagnostic methods improved microorganisms identification from pus aspirates, compared to conventional culture techniques^8^, ^10^ thereby supporting a possible role of PCR microbiology in future.

It is noteworthy that the expert clinical input from the medical microbiologist, applied to the result from traditional culture and sensitivity, was highly valued, and hence the ‘more detailed information’ potentially available from molecular microbiology was not seen as sufficiently meaningful to allow substitution. This was despite the traditional C&S taking sometimes 4 or 5 days to become available (in contrast to the promised speed of molecular processing), given the need to grow the samples in the laboratory and have expert reporting on the results of culture and sensitivity. This approach to the two types of sample processing may be unique to this patient group given that empirical antimicrobials (a locally determined combination of agents to cover the prevalent problematic organisms within the DFU population) are usually initiated in diabetic foot ulcer infection to prevent spreading infection and potential amputation. This is in contrast to other soft‐tissue infection contexts, where the concerns with respect to prevention of antimicrobial resistance usually determine that culture and sensitivity are awaited before mild or moderately infected skin, ulcers or wounds are treated with tailored antimicrobials. Hence, the concurrent approaches of ‘empirical antimicrobials’ and ‘characterisation of organisms’ means that DFU management, in terms of choice of sample approach (tissue or swab), and processing technique (C&S or molecular) might not be replicated elsewhere, for example, if molecular approaches could provide swift results to guide early and narrow spectrum antimicrobial therapy.

Potential limitations in this study are the relatively small sample size and the fact that all participants were medical consultants (doctors rather than nurses or podiatrists). We planned to interview a cross‐section of health professionals but no nurses or podiatrists came forward. None of the participants described being familiar with PCR technology in their current practice.

One strength of the study is that participants belonged to different Hospital Trusts across England and were specialists in different fields associated with the clinical management of DFUs (e.g., microbiology, diabetology, surgery and infectious diseases), therefore providing a good spread in terms of variability in clinical practice due to geographical location and medical specialisation. It would be useful to repeat this work with podiatry and nursing professionals involved in diabetic foot ulcer management to understand whether the attitudes and perspectives differ across professional groups. This is important in light of the increased potential use of non‐medical prescribing to support clinical management and the potential for care decisions regards sampling, processing and prescribing to vary by profession.

## 5 CONCLUSIONS

In conclusion, this study shows that overall, these medical consultants preferred tissue samples over swabs, for microbiological sampling, but reported using the latter when circumstances dictated, such as when there was no access to podiatry expertise. These clinicians thought that PCR microbiology tests could help improve current diagnostic processes for infected DFUs. However, to be clinically useful, PCR reports would need to be based on good clinical samples and reports include information regarding antibiotic sensitivity and guidance on antimicrobial treatment, or be fully discussed in the multi‐disciplinary team (MDT). Clinicians were not yet ready to replace culture and sensitivity with molecular techniques until the sampling, reporting and clinical effectiveness elements were established.

## FUNDING INFORMATION

This research was funded by the UK Health Technology Assessment (HTA) Programme (NIHR HTA ref 163/16/04). The views expressed are those of the author(s) and not necessarily those of the NIHR or the Department of Health and Social Care.

## CONFLICT OF INTEREST STATEMENT

EAN, JEN, FG, JEN, DAR, ALG, SRM and EdiM declare that they have no conflicts of interest.

## Data Availability

The data that support the findings of this study are available from the corresponding author upon reasonable request.
